# Diagnosis, Characterization, and Treatment of Emerging Pathogens, Second Edition

**DOI:** 10.3390/microorganisms13112586

**Published:** 2025-11-13

**Authors:** Shengxi Chen

**Affiliations:** Biodesign Center for Bioenergetics, Arizona State University, Tempe, AZ 85287, USA; shengxi.chen.1@asu.edu

Emerging and re-emerging pathogens continue to pose significant challenges to global health, because of the limits of current diagnostic, therapeutic, and preventive strategies [1,2,3,4,5]. Building upon the success of the first collection on this topic (contribution 1), the second volume of the Special Issue, “Diagnosis, Characterization, and Treatment of Emerging Pathogens, Second Edition” presents a comprehensive selection of original studies and case reports that advance our understanding of pathogenic mechanisms, molecular characterization, innovative diagnostic approaches, and the clinical management of infectious diseases. In summary, these contributions underscore the ongoing need for interdisciplinary collaboration that bridges microbiology, molecular biology, clinical medicine, and public health to effectively confront the evolving landscape of infectious threats.

In this Special Issue, tuberculosis (TB) remains a central focus, reflecting its ongoing significance as a leading cause of infectious mortality worldwide. Wumaier et al. investigate the role of G-protein-coupled receptor 84 (GPR84) in the interaction between *Mycobacterium tuberculosis* (Mtb) and macrophages (contribution 2). Their findings reveal that GPR84 modulates pro-inflammatory cytokine expression and promotes lipid droplet accumulation, thereby facilitating bacterial persistence within host cells. This work provides new insight into host–pathogen interactions and identifies GPR84 as a potential therapeutic target for host-directed TB treatment strategies. Complementing this, Cuthbert et al. report the structural characterization of the Mtb encapsulin in complex with dye-decolorizing peroxidase (DyP) (contribution 3). The study presents high-resolution crystallographic data that elucidate the structural features of this encapsulated complex and its relevance to oxidative stress resistance, adding an important layer to our understanding of Mtb’s adaptive mechanisms within macrophages.

Technological advancements in microbial diagnostics are also well represented. Handigund and Lee evaluate the Novaplex™ multiplex real-time PCR assay for the detection of *Streptococcus agalactiae* serotypes, demonstrating its accuracy, efficiency, and potential utility for routine clinical use in serotype identification (contribution 4). Chen et al. further contribute to diagnostic innovation through the integration of artificial intelligence and machine learning into an automated microscopy platform for acid-fast bacilli detection in sputum smears (contribution 5). Their results show that this intelligent microscopy system achieves high sensitivity and specificity, offering a promising approach for improving diagnostic throughput in TB laboratories, particularly in high-burden settings.

Viral and fungal pathogens are also addressed through molecular and epidemiological investigations. Tikute et al. describe the emergence of the recombinant Coxsackievirus A6 subclade D3/Y during a hand–foot–and–mouth disease outbreak in India, underscoring the rapid genomic evolution and epidemiological dynamics of enteroviruses (contribution 6). Similarly, van Steen et al. present a fatal case of necrotizing pneumonia caused by exfoliative toxin etE2-producing *Staphylococcus aureus* belonging to sequence type ST152, highlighting the pathogenic potential of emerging *S. aureus* lineages harboring novel virulence factors (contribution 7). In a parallel line of inquiry, Stolfa et al. report the first case of *Candida auris* sepsis in Southern Italy, integrating antifungal susceptibility testing and whole-genome sequencing to characterize this multidrug-resistant yeast (contribution 8). Their findings emphasize the importance of genomic surveillance and stringent infection control measures in preventing nosocomial transmission.

In the field of pediatric infectious diseases, Di Meglio et al. provide two case reports and a systematic review of *Streptococcus pyogenes* meningitis, a rare but severe condition (contribution 9). Their analysis identifies key prognostic indicators, including bacteremia and a high Phoenix Sepsis Score, which are associated with poor outcomes. This work contributes valuable clinical evidence to guide management and prognosis in pediatric patients. Lastly, Cheng and Chen review the application of in vitro models for studying the complex interactions between environmental exposures and the human microbiota (contribution 10). Their review highlights how these systems can serve as ethically viable and mechanistically informative tools to investigate exposure–microbiota dynamics, offering an important framework for future research on environmental determinants of microbial ecology and human health.

Collectively, the studies presented in this Special Issue illustrate the multidimensional progress being made in pathogen diagnosis, molecular characterization, and treatment innovation. From molecular insights into host–pathogen interactions to the development of advanced diagnostic platforms and genomic surveillance tools, these contributions underscore the importance of cross-disciplinary collaboration in addressing the ongoing and emerging challenges posed by infectious diseases.

## Data Availability

No new data were created or analyzed in this study. Data sharing is not applicable to this article.
